# Investigating the Impact of Scenario‐Based Moral Concepts Training on the Professional Moral Courage of Nursing Students: A Protocol for a Randomized Controlled Trial

**DOI:** 10.1002/hsr2.71021

**Published:** 2025-07-13

**Authors:** Ali Akbari, Arvin Mirshahi, Fatemeh Mehravar, Ali Karimi Rozveh, Alireza Nikbakht Nasrabadi

**Affiliations:** ^1^ Department of Medical‐Surgical Nursing, School of Nursing and Midwifery Tehran University of Medical Sciences Tehran Iran; ^2^ Department of Medical‐Surgical Nursing, School of Nursing and Midwifery, Students' Scientific Research Center Tehran University of Medical Sciences Tehran Iran; ^3^ Center for Palliative and Supportive Care University of Alabama at Birmingham Birmingham Alabama USA; ^4^ Department of Biostatistics and Epidemiology, School of Health, Ischemic Disorders Research Center Golestan University of Medical Sciences Gorgan Golestan Iran

**Keywords:** ethical concepts, moral concepts, moral courage, nursing students, scenario‐based training

## Abstract

**Background and Aims:**

Moral courage is vital in nursing, enabling professionals to make ethical decisions and uphold values, even in challenging situations. Nursing students often struggle with moral decision‐making due to limited experience and insufficient ethical training. This study proposes a randomized controlled trial (RCT) to evaluate the effectiveness of scenario‐based ethics training compared to traditional methods in improving professional moral courage among nursing students.

**Methods and Analysis:**

This RCT involved a 3‐month scenario‐based ethics training program delivered under faculty supervision, targeting sixth‐semester undergraduate nursing students. Participants (*n* = 48) were randomly assigned to either the intervention group or the control group. Both groups received traditional ethics education, however, the intervention group also participated in two workshops focused on core and derived ethical concepts (e.g., autonomy, confidentiality) utilizing a scenario‐based approach. This was followed by weekly online follow‐ups and discussions conducted via WhatsApp Messenger. The outcomes will be assessed using the Sekerka Professional Moral Courage Questionnaire at four time points: immediately before, immediately after, and at 1 and 3 months postintervention. The questionnaire has demonstrated acceptable validity and reliability in previous studies, with Cronbach's *α* exceeding 0.8, and a value of 0.977 reported in an Iranian validation study. In this study, face validity was confirmed by ten nursing faculty members. The data will be analyzed using *t*‐tests, *χ* ^2^ tests, and ANCOVA to evaluate changes in professional moral courage (SPSS v22, *p* < 0.05).

**Conclusion:**

This protocol outlines a trial to evaluate the impact of scenario‐based training on enhancing nursing students' professional moral courage and improving ethics education in clinical settings.

**Trial Registration:**

Iranian Clinical Trials Registry: IRCT20240319061338N1.

## 1 Introduction

Ethics is an essential and inseparable part of human life, addressing behaviors, ideal ways of living, and distinguishing between good and evil [1]. With the increasing complexity of life, ethical challenges have also become more prevalent, leading to negative consequences for mental health [2]. Nurses, especially in care settings, frequently face ethical dilemmas, and factors such as staff shortages, organizational misalignment, and managerial weaknesses exacerbate these challenges [3, 4]. These issues can lead to burnout and even result in nurses leaving the profession [5].

Nursing students also require skills in ethical analysis and decision‐making to cope with the expectations and complexities of clinical settings [6, 7]. However, many of them find dealing with moral distress difficult due to fear of taking ethically appropriate actions [8]. To overcome these challenges, they need moral courage, which entails commitment to principles and the willingness to accept risks in ethical decision‐making [9, 10].

Moral courage is a fundamental concept in the nursing profession, helping nurses provide valuable and professional care to patients and society while embodying desirable human qualities [11]. Moral courage entails making decisions and taking actions based on ethical values, even in challenging situations. This virtue is essential for healthcare professionals to overcome ethical conflicts and professional challenges with a clear conscience [12]. It bridges the gap between awareness of professional values and commitments and their implementation, even in the face of risks such as social rejection and shame, allowing individuals to accept vulnerability to uphold their values [13].

According to Sekerka and colleagues, moral courage is adherence to ethical principles when faced with challenges and confronting moral corruption, particularly in making difficult decisions under challenging circumstances [14]. It is considered a multidimensional construct, involving ethical sensitivity, emotional resilience, and the behavioral readiness to act upon one's values despite potential risks. Rather than being a fixed personality trait, moral courage encompasses a set of professional attitudes and competencies that can be nurtured and strengthened through experience and targeted ethical education [14]. Nurses must be familiar with ethical issues to perform professionally, and this sensitivity should be emphasized during their education [15]. Although ethical training is included in the nursing curriculum, mainly through the “Professional Ethics and Communication Skills” course delivered via lectures, this traditional training is often insufficient to prepare students for the ethical challenges of clinical practice [6]. A lack of clinical experience, low self‐confidence, and inadequate education diminish students' capacity for moral courage and gradually reduce their ethical sensitivity [16, 17].

Nursing students need specific skills to effectively cope with moral distress, and nursing educators are responsible for providing learning experiences that prepare them to face ethical issues [6]. Teaching ethics to students fosters a deeper understanding of perspectives and their effective participation in addressing ethical challenges after graduation. For this reason, ethical principles are considered a cornerstone of the nursing curriculum [18]. Some studies suggest that teaching moral concepts enhances moral sensitivity; however, traditional teaching methods, such as lecture‐based and rule‐oriented instruction, primarily focus on the transmission of theoretical content and professional codes, with limited emphasis on critical thinking or real‐life application. As a result, they may fail to adequately prepare students for ethical decision‐making in complex clinical environments [19, 20, 21].

Scenario‐based training, as an interactive approach, enhances students' decision‐making skills and prepares them to face ethical challenges in difficult situations [21, 22]. While previous research has focused on issues like moral distress and moral courage among nurses, few studies have examined these issues among nursing students. Understanding the factors that influence students' moral courage can help healthcare and educational stakeholders design appropriate interventions to enhance moral courage among students [22, 23].

Innovative teaching methods, such as scenario‐based training, which remains limited in Iran, may potentially improve students' ethical skills and abilities. This approach can guide students toward a deeper understanding of ethical concepts, strengthen professional behaviors, and foster an ethical culture in educational and professional settings [21]. By presenting realistic scenarios, it is possible to enhance students' moral resilience against the pressures and challenges of the healthcare system [24].

Based on the literature review, moral courage is essential for professional decision‐making in the nursing profession; however, traditional teaching methods fail to prepare students to address ethical challenges adequately. Scenario‐based training may have the potential to enhance students' ethical competencies. Despite the importance of this issue, to the best of our knowledge, no study has yet examined the impact of scenario‐based training on nursing students' professional moral courage. This study aims to fill this gap by investigating how scenario‐based training of moral concepts compares with traditional teaching methods. Accordingly, this study aims to investigate the impact of scenario‐based moral concepts training on the professional moral courage of nursing students, with the hypothesis that scenario‐based ethics training will significantly increase the professional moral courage of nursing students compared to traditional teaching methods.

## 2 Methods/Design

### 2.1 Study Design

The present study is a randomized controlled trial (RCT) conducted at a single site and in an open‐label format. The study involves two groups: a control group and an intervention group, aiming to investigate the impact of scenario‐based training on moral concepts on the professional moral courage of nursing students. This protocol has been developed in accordance with the 2022 Standard Protocol Items: Recommendations for Interventional Trials (SPIRIT) guidelines [25].

### 2.2 Study Setting

The study will be conducted at the School of Nursing and Midwifery, Tehran University of Medical Sciences. As one of the primary nursing education centers in Iran, this school provides an appropriate environment for implementing the research.

### 2.3 Eligibility Criteria

The nursing students enrolled in the School of Nursing and Midwifery will be assessed based on the following eligibility criteria.

#### 2.3.1 Inclusion Criteria

Participants will be included in the study if they meet the following conditions: (1) willingness to participate in the study, (2) successful completion of the theoretical course on professional ethics, (3) at least 1 year of clinical experience as a nursing student (internship or externship), and (4) access to and ability to use a smartphone and the internet.

#### 2.3.2 Exclusion Criteria

Participants will be excluded or withdrawn from the study if they: (1) have been diagnosed with psychological or cognitive disorders, (2) have previously participated in similar ethical empowerment workshops, and (3) fail to attend one of the scheduled ethics workshops in the program.

Information regarding psychological or cognitive disorders will be obtained solely through voluntary self‐reporting by participants during the eligibility screening process. No medical records will be accessed, and responses will be kept strictly confidential by the researcher in accordance with ethical approval protocols.

### 2.4 Interventions

The present intervention involves a 3‐month program led by a Master's degree nursing student under the supervision of a nursing faculty member specializing in ethics education. Both groups will receive traditional training based on the professional ethics and relationships course included in the nursing curriculum, typically taken in the second semester. This course covers topics such as ethical principles, communication skills, and professional behavior.

The intervention group, in addition to traditional training, will participate in a specialized, scenario‐based workshop designed to deepen their understanding of moral concepts, strengthen their moral courage, and develop the skills and confidence needed to address complex ethical challenges. This approach immerses students in realistic clinical scenarios, enabling them to better grasp ethical principles and apply them effectively in practice.

**Figure 1:**
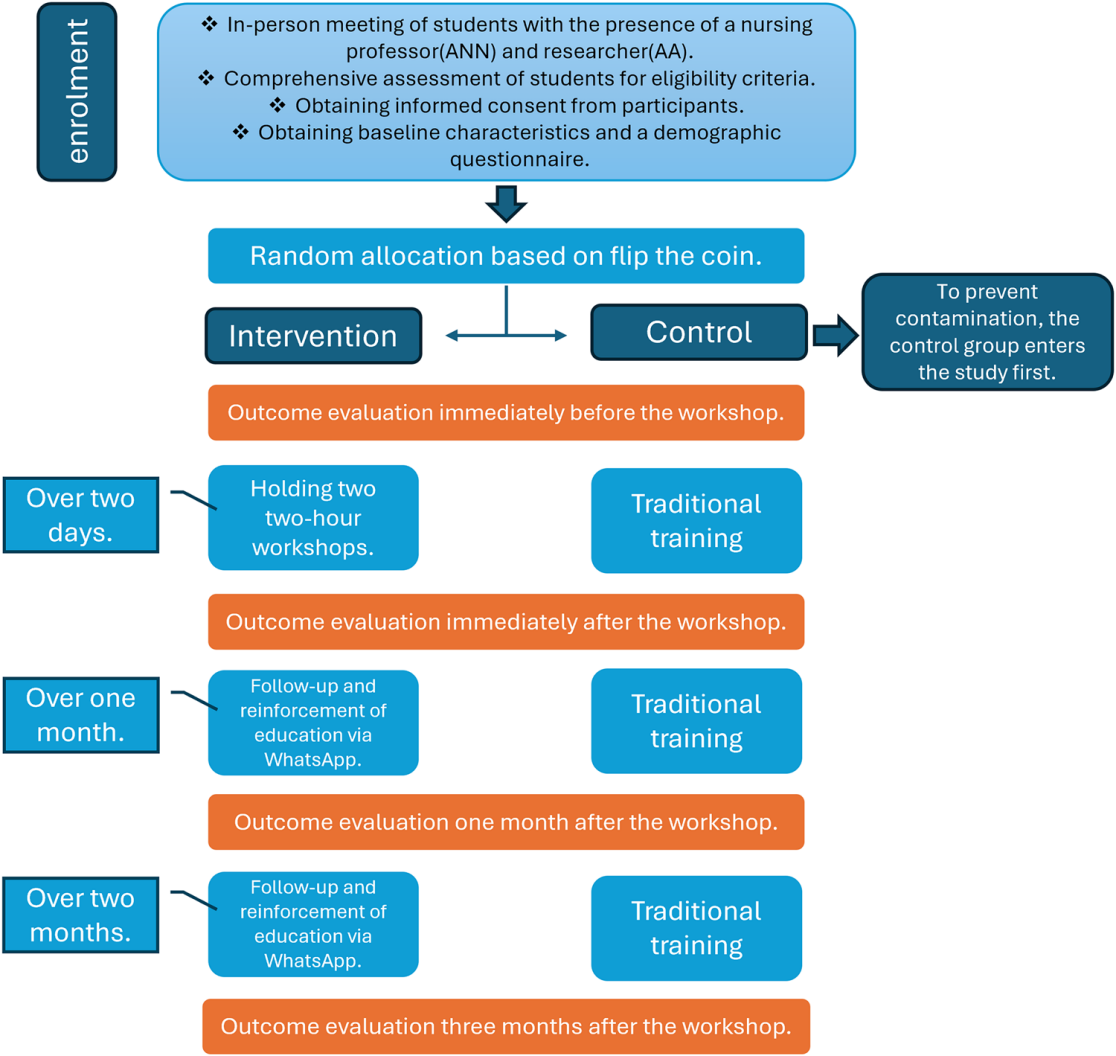
Schematic representation of the study design.

The intervention consists of two workshop sessions, online education, and follow‐ups over 3 months. After obtaining written consent, both groups will complete a demographic questionnaire. Figure 1 illustrates a schematic representation of the study design.

#### 2.4.1 Scenario‐Based Moral Concepts Workshop for the Intervention Group

A moral concepts workshop will be held for the intervention group. At the beginning of the first workshop, after obtaining students' phone numbers, a collaborative group will be created on an internet‐based social media platform (WhatsApp). This group will include the researcher and the nursing ethics professor from the School of Nursing, Tehran University of Medical Sciences, to enhance the training, provide long‐term follow‐ups, and monitor the students in various aspects. The intervention group will receive the educational program on moral concepts through two 2‐h workshops over 2 consecutive days. In the first workshop, the basic principles of medical ethics will be discussed, and in the second, concepts derived from these principles will be analyzed.

The pedagogical approach used in this training intervention was based on constructivist and experiential learning models. This approach emphasizes active participation, collaborative problem‐solving, and reflective thinking. Through scenario‐based learning, students are exposed to complex ethical dilemmas similar to those encountered in real clinical settings, allowing them to apply theoretical knowledge in a practical, decision‐oriented environment. The intervention was designed to encourage critical thinking, moral reasoning, and confidence in ethical decision‐making.

The curriculum of the training was structured around the four core principles of biomedical ethics—autonomy, beneficence, nonmaleficence, and justice—as well as key derived ethical concepts such as confidentiality, honesty, disclosure, and moral courage. These concepts were selected based on the foundational biomedical ethics framework proposed by Beauchamp and Childress, and the professional codes of ethics developed by the American Nurses Association (ANA) and the International Council of Nurses (ICN). Their contextual relevance and educational priority were confirmed through expert review by ten faculty members specializing in nursing ethics at Tehran University of Medical Sciences.

All educational materials, including the ethical scenarios, were developed by the research team and underwent face and content validity review by ten faculty members with expertise in nursing ethics at Tehran University of Medical Sciences. This review process ensured that the scenarios reflected realistic, ethically significant clinical challenges. The training methodology integrated small group discussions, interactive scenario analysis, and weekly follow‐up activities, all designed to foster ethical sensitivity and professional growth among participants.

The workshops were cofacilitated by a nursing faculty member specializing in ethics education and a trained research assistant in nursing, ensuring both academic rigor and interactive student engagement. The faculty member led the educational discussions and scenario analyses, while the assistant coordinated the sessions and provided ongoing support throughout the training period.

During the workshop sessions, as well as while reviewing and analyzing the scenarios, both the researcher and a faculty member specializing in ethics will be present to answer any questions that may arise. In the intervention, the researcher and the faculty member, an expert in teaching ethics, will actively participate in all in‐person sessions, ensuring comprehensive guidance and support for the participants. Table 1 refers to the details of the ethics workshop.

**Table 1:** Workshop topics details table in two sessions.

Activity headings	Details covered	Time schedule
*First Workshop: Training on Core Principles of Medical Ethics (2 h)*		
Introduction to Ethical Principles	The workshop began with an introduction to the core principles of medical ethics, including Beneficence, Nonmaleficence, Justice, and Autonomy. The instructor explained each principle, its importance in professional decision‐making, and used clinical examples for better understanding.	20 min
Group Case Analysis	Students were divided into small groups, with each group receiving a clinical case related to an ethical principle. Groups identified ethical dilemmas and proposed solutions aligned with the discussed principles. The results of their analyses were then presented to the larger group.	30 min
Group Discussion and Feedback	All groups shared their results, followed by a general discussion on different decisions. The instructor facilitated the session by highlighting key points and exploring various solutions.	20 min
Analysis of Complex Cases	Groups analyzed more complex scenarios involving multiple ethical dilemmas. They had to select the best solutions while considering various conditions and explain how ethical principles influenced their decisions.	40 min
Summary and Feedback	The instructor provided feedback on the analyses and emphasized critical points students should consider in real‐life situations.	10 min
*Second Workshop: Training on Concepts Derived From Ethical Principles (2 h)*		
Introduction to Derived Ethical Concepts	This session introduced ethical concepts such as Confidentiality, Honesty, Disclosure, and Moral Courage. The instructor explained their connection to core ethical principles and their application in clinical decision‐making.	20 min
Advanced Case Analysis	Groups received cases involving multiple conflicts between ethical principles and concepts. They analyzed these cases and proposed solutions that balanced various ethical values.	40 min
Group Review and Critique	Each group presented their analysis to the class, and other students critiqued their work. This session enhanced critical evaluation and analytical thinking skills.	30 min
Experience Sharing and Open Discussion	Students shared their experiences and perspectives on similar cases encountered in clinical settings. These discussions deepened understanding and practical application of taught concepts.	20 min
Final Summary and Feedback	The instructor provided final feedback on students' performances and highlighted key areas for improving their ethical skills.	10 min

The ethical scenarios presented in the workshops will have characteristics such as realism, challenge, moral significance, ambiguity, value conflicts, feedback responsiveness, flexibility, and an emphasis on active decision‐making [26]. These features are crucial in ethics education as they help students familiarize themselves with complex and common situations in the nursing profession, deepening their understanding of ethical principles. Furthermore, the presence of ethical conflicts and ambiguity in the scenarios enhances students' critical thinking and decision‐making skills and, through feedback and outcome analysis, provides an opportunity for the improvement of their ethical competencies [21, 22].

#### 2.4.2 Postworkshop Educational Support and Follow‐Up

After the workshops, the materials taught to the students, including the prepared slides, ethical scenarios, and the topics discussed in the sessions, will be shared on the WhatsApp group to ensure easy access and continuous review of the content. Additionally, on the second day after the completion of the workshop, a comprehensive training booklet will be provided to the students. This booklet will include the principles and concepts of ethics, practical examples, and supplementary explanations, serving as a thorough resource for review and deeper learning. Notably, the booklet was approved by five faculty members who specialize in teaching ethics and have expertise in the field of ethics. These actions are designed to strengthen the learning process and ensure students can continue to review the educational content even after the workshops conclude.

Students will be encouraged to engage in discussions and exchange ideas about the educational content through the WhatsApp group, allowing them to raise questions, which will help sustain the learning process and deepen their ethical knowledge. The WhatsApp group will provide students with the opportunity to share their questions and opinions regarding the scenarios presented in the workshops and resolve any ambiguities. This communication platform, with its focus on ethical scenarios, will enable students to analyze and review the issues raised during the workshops and explore different solutions to the ethical challenges posed in the scenarios.

The WhatsApp discussions were moderated by the research assistant with supervision from the ethics faculty member. These discussions aimed solely to reinforce educational content and promote student reflection. No qualitative data from these interactions were analyzed, recorded, or used in the outcome assessment, to protect participant confidentiality and focus the analysis on the primary quantitative outcome.

Furthermore, discussing various experiences and perspectives related to the scenarios will help students gain a deeper understanding of ethical principles and their application in real‐world situations. This scenario‐based approach will contribute to the continuation of learning and the enhancement of their ethical decision‐making skills [27, 28].

The scenario‐based approach will be designed in such a way that in both workshops, the analysis of ethical cases will play a central role. This method is expected to strengthen students' ethical reasoning and confidence in moral action, as reflected by improvements in their professional moral courage. All the educational materials and scenarios presented during the workshops will be shared in the WhatsApp group after the sessions to facilitate further review and analysis.

The interaction among students in the WhatsApp group will continue, allowing them to share their analyses and opinions on new scenarios. Through the exchange of knowledge and experiences, students will be able to further enhance their learning.

This structure, by increasing the stages and focusing on deeper analyses, will aid in improving the students' analytical and decision‐making skills, thereby strengthening their preparation to face ethical challenges in real‐world settings [27].

After the completion of the workshops, the intervention group students will be followed up weekly for 3 months to reinforce and consolidate the training provided, with a focus on ethical scenarios. During these follow‐ups, new ethical scenarios will be sent each week for the students to analyze and evaluate. Additionally, videos and segments from educational films, designed based on similar scenarios, will be provided to strengthen students' understanding of ethical challenges and how to address them.

This scenario‐based approach will allow students to consistently encounter complex ethical situations and improve their decision‐making skills. Throughout this period, any questions and uncertainties related to the scenarios will be addressed in the WhatsApp group, where students will be encouraged to share their opinions and analyses of the scenarios and the ethical challenges presented. This emphasis on scenarios will ensure that students' active and continuous learning is maintained, enhancing their practical and applicable understanding of ethical concepts [26].

The SPIRIT 2013 template [29] describes the study schedule enrollment, interventions, and assessments (Table 2).

**Table 2:** Schedule for enrollment, interventions, and assessments in this study.

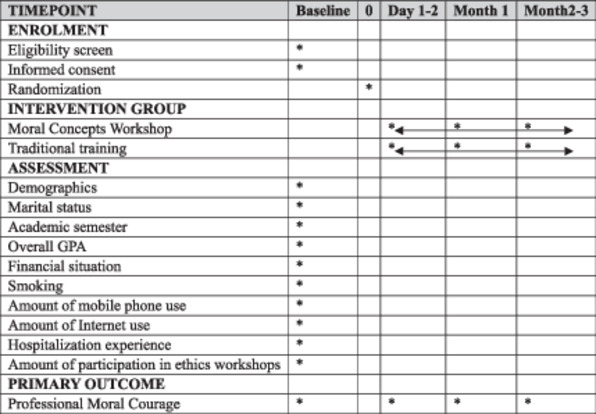

*Note:* Background variables were collected to describe the sample characteristics and to identify potential confounders that may influence ethical reasoning and moral courage. “Overall GPA” refers to the participants' cumulative grade point average at the time of enrollment in the study.

#### 2.4.3 Traditional Training

The students in the control group will not receive the scenario‐based intervention program designed for the intervention group. However, they will receive the traditional training in ethics routinely offered at the university, which consists of the “Professional Ethics and Communication Skills” course. This course is part of the standard nursing curriculum, typically delivered during the second semester, and is presented through lectures. It covers essential topics such as ethical principles (e.g., beneficence, nonmaleficence, autonomy, justice), communication strategies, and professional behavior. The content is primarily theoretical, instructor‐centered, and does not include interactive or scenario‐based activities. Meanwhile, the students in the intervention group will receive, in addition to this traditional training, a scenario‐based ethics training program. Furthermore, all educational materials related to the intervention will be provided to the control group after the study is completed and after the primary outcome variable has been measured.

### 2.5 Outcome Measure

The effectiveness of the 3‐month intervention will be evaluated through the following outcome variables.

#### 2.5.1 Primary Outcome

The primary outcome of this study will be:

*The professional Moral Courage of Nursing Students will be assessed by the Professional Moral Courage Questionnaire.*

The Professional Moral Courage Questionnaire [14] is used to measure moral courage in professional and workplace environments. It consists of 15 items across five dimensions: Ethical Principles, Multiple Values, Risk or Threat Tolerance, Beyond Compliance, and Ethical Goals, rated on a 7‐point Likert scale. Scores range from 15 to 105, with classifications of *Low* (< 50), *Moderate* (50–70), and *High* (> 70) Moral Courage.

The validity and reliability of the tool have been confirmed through previous studies. Construct and content validity were established by Sekerka et al. [14], and the tool demonstrated high internal consistency, with a Cronbach's *α* exceeding 0.8.

In the Iranian population, Hatami and Naderi Beni verified the validity and reported a Cronbach's *α* of 0.977 [30]. Before the study face validity of the tool will be reviewed by ten faculty members from Tehran University of Medical Sciences.

### 2.6 Data Collection

In this study, data will be collected using two questionnaires: a demographic information questionnaire and the Professional Moral Courage questionnaire by Sekerka et al. [14]. The demographic information of the students will be gathered through a written questionnaire at the beginning of the study. Data on the dependent variable (Professional Moral Courage) will be collected immediately before the workshop, immediately after the workshop, 1 month later, and 3 months after the intervention. Both the intervention and control groups will be assessed for this variable, and they will complete the standardized Professional Moral Courage questionnaire by Sekerka et al. [14] in person using printed paper and pencil.

To prevent information leakage and sample contamination, the control group will complete the study first, followed by the intervention group.

### 2.7 Sample Size

The sample size for this study was calculated using G*Power software (version 3.1.9.4), based on the standard formula for comparing means between two independent groups:

$$n={\left(Z(\alpha /2)\right)+Z_{\beta })}^{2}\cdot \left(\sigma_{1}^{2}+\sigma_{2}^{2}\right)/\Delta^{2}.$$

The calculation was informed by the findings of a previous study conducted by Hosseini Yazdan Abad Sofla et al. [31], which reported an effect size of 1.18. Using a significance level of 0.05 and a statistical power of 0.95, the required sample size was estimated at 40 participants. To account for an anticipated 20% attrition rate, the sample size was increased to 48 participants, ensuring adequate power to detect the expected effect even with potential dropouts.

### 2.8 Recruitment

To conduct this study, the researcher will first obtain permission from the Joint Ethical Committee of the School of Nursing and Midwifery and the School of Rehabilitation at Tehran University of Medical Sciences, as well as permission from the Dean of the School of Nursing and Midwifery. The researcher will then visit the School of Nursing and Midwifery to select samples based on the inclusion criteria. All undergraduate nursing students from 6th semester, which includes two classes, will be considered for the study. Eligible participants will be selected through a census. A list of students will be obtained from the school administration, and students will be invited to participate by the principal investigator during an in‐person meeting. After explaining the study's aims and methods, informed consent will be obtained from all participants. The classes will be randomly assigned to either the intervention group (Class A) or the control group (Class B) using simple randomization. In this phase, further explanations about the study will be provided, and students will be encouraged to follow the study's timeline. To minimize the risk of contamination between the classes and prevent information leakage between the two groups, the control group will be included in the study first, followed by the intervention group.

### 2.9 Random Allocation

The allocation of classes to the intervention or control group will be done using simple randomization, specifically through a coin flip. One class will be assigned to the “heads” side, and the other to the “tails” side. After flipping the coin, the classes will be assigned to either the intervention group (Class A) or the control group (Class B).

### 2.10 Blinding

Due to the nature of this intervention, it will not be possible to blind the participants or the intervening party. However, the outcome assessor will work independently from the interventionist and will not know which group the participants belong to. The outcome assessors, data entry personnel for SPSS, and epidemiological consultants will be blinded and trained to ensure they do not inquire about whether participants have received the intervention. Additionally, participants will be instructed not to discuss their activities with the outcome assessors.

### 2.11 Data Management

The demographic questionnaire will be completed by both the control and intervention groups after obtaining written consent. The intervention and control groups will be assessed on the dependent variable (Professional Moral Courage) immediately before the training workshop, immediately after its completion, 1 month later, and 3 months after the intervention. The standardized Professional Moral Courage Questionnaire by Sekerka et al. [14] will be completed in person and printed format. To minimize and prevent data loss, students will be encouraged and monitored to complete the questionnaires fully at each stage. Additionally, the outcome assessor will receive training to ensure the accurate and complete collection of data. Data entry into SPSS version 26 will be performed by a trained researcher, and the accuracy of the entered data will be verified by another member of the research team. To maintain participant confidentiality, the data will be stored using anonymous code numbers. Finally, data analysis will be conducted by an epidemiologist.

### 2.12 Statistical Analysis

The statistical analysis plan and reporting approach in this protocol follow the Statistical Analyses and Methods in the Published Literature guidelines (SAMPL) to ensure transparency, accuracy, and consistency in the presentation of all statistical information. To describe quantitative variables, mean and standard deviation will be utilized, while frequency tables, including percentages, will be employed for qualitative variables. The Kolmogorov–Smirnov test will be used to assess the normality of the data distribution. To compare quantitative and qualitative variables between the two groups before the intervention, the independent *t*‐test and *χ* ^2^ test will be applied, respectively. Additionally, the paired *t*‐test will be used to evaluate the effectiveness of the intervention in the control and intervention groups. Analysis of covariance (ANCOVA) will be conducted to control for the effects of confounding variables. Data analysis will be performed using SPSS software version 22, and a significance level of *p* < 0.05 will be considered statistically significant for all tests.

## 3 Discussion

Professional ethics is one of the key elements in improving the quality of nursing services and plays a crucial role in addressing ethical challenges in the workplace [2, 3]. Scenario‐based training focusing on moral concepts is an innovative approach that empowers nursing students by simulating real‐life situations, thus preparing them for ethical decision‐making [21, 22].

This protocol outlines the design of a RCT to investigate how this type of education impacts the development of professional moral courage in nursing students. It highlights the importance of this approach in enhancing the quality of education and preparing professional healthcare personnel.

Scenario‐based training, as an innovative educational method, emphasizes learning through the simulation of real‐life and interactive situations. By providing opportunities to face simulated challenges, this approach can help students enhance their decision‐making, critical thinking, and ethical analysis skills [32]. In nursing, where students encounter complex ethical dilemmas, scenario‐based training serves as a valuable tool for preparing them for professional situations. While various studies, such as those by Errington [33] and Thistlethwaite et al. [34], have demonstrated the positive effects of this method on ethical learning skills, discrepancies in reported outcomes suggest that differences in scenario design and educational contexts may influence the results.

In ethical education, designing scenarios aligned with real‐world challenges allows students to apply theoretical concepts in practice and analyze complex situations. Specifically, facing conditions that require difficult decisions can profoundly impact students' confidence and sense of responsibility [35]. Studies such as Hege et al. [36] have emphasized that scenario‐based training can effectively strengthen ethical skills. However, it is essential to note that the effectiveness of scenario‐based training may vary depending on students' prior knowledge, experience, and the educational environment [36, 37].

In conclusion, scenario‐based training provides a suitable framework for ethical learning and professional decision‐making by simulating real‐life situations and offering a safe environment to test various strategies. Nonetheless, examining different contexts reveals that the implementation conditions of this method and the precise design of scenarios play a significant role in its success [38, 39].

It is anticipated that scenario‐based ethical education will enhance the professional moral courage of nursing students. Through this approach, students will be able to face ethical challenges in simulated scenarios and make informed decisions that boost their confidence and moral courage in professional environments. The goal of this intervention is to demonstrate that scenario‐based training positively impacts students' ethical competencies and strengthens their moral courage in real‐life situations.

Although the training program addresses multiple educational dimensions, moral courage was selected as the primary outcome due to its integrative nature and strong connection to ethical performance in clinical practice. It reflects not only knowledge and attitudes but also the readiness to act ethically under pressure, which makes it a suitable and measurable indicator of overall ethical development.

Like any other study, this study is not without limitations. Despite efforts to minimize them, certain constraints may exist. One limitation is the inability to conduct in‐person follow‐ups. Consequently, student follow‐ups and reinforcement of educational content were carried out online and remotely. While this virtual follow‐up method may restrict close interactions and direct observation of students' progress, it facilitates continuous access to education and sustained learning. Additionally, due to the nature of the intervention, the study will not be blinded to participants. This could potentially influence the results, although outcome assessors and the epidemiology consultant will remain blinded.

This study assumes that scenario‐based training can improve nursing students' professional moral courage. If the findings of this study are positive, this educational approach could serve as an effective tool for fostering nursing students' moral courage in Iran and other countries, ultimately leading to enhanced professional performance and ethical competencies in real‐life situations.

## Author Contributions

**Ali Akbari:** writing – original draft, writing – review and editing, conceptualization, methodology, investigation. **Arvin Mirshahi:** writing – review and editing, investigation. **Fatemeh Mehravar:** writing – review and editing, data curation, statistical analysis, formal analysis. **Ali Karimi Rozveh:** writing – review and editing, visualization. **Alireza Nikbakht Nasrabadi:** supervision, validation, methodology, project administration, overall responsibility for the project. All authors have read and approved the final manuscript.

## Ethics Statement

Ethical approval for the study protocol was obtained from the Research Ethics Committee of the School of Nursing and Midwifery at the Tehran University of Medical Sciences (TUMS) on March 17, 2024 with reference number [IR.TUMS.FNM.REC.1402.249]. The protocol has also been registered with the Iranian Clinical Trials Registry (IRCT) under the identifier [IRCT20240319061338N1]. To ensure ethical principles are followed, all educational materials from the empowerment program, along with an electronic version of the study materials, will be sent to the control group students after the completion of the study and data collection. The objective of this study is to present the findings to relevant stakeholders and publish the results in reputable academic journals.

## Consent

Written consent will be obtained from all students, and they will be fully informed of their rights to voluntary participation and the ability to withdraw at any stage of the study. Data will be stored confidentially using anonymous codes. The contact information of the researcher will be provided to the students in case they have any questions or need guidance.

## Conflicts of Interest

The authors declare no conflicts of interest.

## Student and Public Involvement

There was no direct public or student involvement in the development of this protocol. However, a group of nursing students voluntarily contributed to the study design by reviewing the educational content and providing feedback based on their educational and clinical experiences.

## Transparency Statement

The lead author Alireza Nikbakht Nasrabadi affirms that this manuscript is an honest, accurate, and transparent account of the study being reported; that no important aspects of the study have been omitted; and that any discrepancies from the study as planned (and, if relevant, registered) have been explained.

## Data Availability

Dr. Alireza Nikbakht Nasrabadi had full access to all of the data in this study and takes complete responsibility for the integrity of the data and the accuracy of the data analysis.
